# Leveraging integrated youth services for social prescribing: a case study of Youth Wellness Hubs Ontario

**DOI:** 10.24095/hpcdp.44.9.02

**Published:** 2024-09

**Authors:** Aaron Turpin, Deb Chiodo, Maria Talotta, Jo Henderson

**Affiliations:** 1 Youth Wellness Hubs Ontario, Toronto, Ontario, Canada; 2 Department of Human Services and Early Learning, MacEwan University, Edmonton, Alberta, Canada; 3 Department of Psychiatry, University of Toronto, Toronto, Ontario, Canada

**Keywords:** social prescribing, integrated youth services, youth services, youth well-being

## Abstract

**Introduction:**

Integrated youth services (IYS) presents a unique opportunity to adopt social prescribing (SP) strategies within the IYS service model by developing and leveraging a highly connected multidisciplinary network of clinical and community-based service providers to tackle health inequities and enhance service access and outcomes for youth. This paper outlines a case study of Youth Wellness Hubs Ontario (YWHO), Canada, a collective of youth-serving organizations integrated and networked, and operating as a learning health system implementing SP services. The main study objective was to document how YWHO hubs engage in social prescribing through service provision.

**Methods:**

We adopted an embedded case study approach. Data were collected from youth (n=6361) aged between 12 and 25 years who were seeking services at a YWHO hub. Descriptive analyses, including frequencies across categories, were generated from service data, including reason for visit, needs addressed and service provided.

**Results:**

A comparative analysis of services requested and provided found that youth across visits to YWHO hubs were engaging with multiple services and service providers, with a wide range of health, mental health and social support needs being addressed.

**Conclusion:**

YWHO implements SP services that aim to improve mental health resilience by supporting the vocational, educational and socialization needs of young people accessing IYS through YWHO hubs.

**Highlights**

- IYS exemplifies an innovative approach to SP through the development of a closely connected network of interdisciplinary service providers.
- Youth engaged in IYS are likely to connect with multiple services concurrently as biopsychosocial needs are identified and addressed.
- The most common services provided by YWHO address mental health, educational and relationships needs, and are provided by mental health workers, care navigators and education or training support workers.

## Introduction

Mental health service use among youth continues to be low,^1^,^2^ while treatment for substance use has been found insufficient for meeting youths’ needs.^3^ Youth seeking services commonly experience multiple internalizing (e.g. depressive symptoms, anxiety, stress) and externalizing (e.g. inattention, substance use, hyperactivity) difficulties^3^-^6^ that can lead to poor developmental outcomes if not fully addressed.^7^ The need for quality integrated services addressing these health disparities concurrently is well documented,^2^,^5^,^8^-^11^ yet service frameworks adopting this approach are largely absent from practice.9 

Addressing this service gap, integrated youth services (IYS) is an innovative care approach that establishes multidisciplinary teams of professionals who work together to meet the co-occurring needs of youth and their families.^4^,^9^,^10^ IYS establishes a shared vision of delivering youth services across an integrated network of providers,^9^,^11^ thereby transcending the capacity of individual programs and leveraging the power of a collective network providing wholistic support and reducing fragmentation of care.^10^-^13^ By enhancing connections to and between services, IYS supports timely and effective health and mental health care for youth,^2^ thereby decreasing health disparities faced by this population.^7^,^8^

A core component of IYS includes leveraging the power of social prescribing (SP) practices for the purpose of enhancing service engagement for youth.^4^,^10^ SP refers to activities that connect service users to person-centred health and mental health services in a community setting as part of an intervention.^14^ These services aim to support individuals in addressing their own social and health needs through community connections^15^ by facilitating referrals from clinical to nonclinical community services.^16^ As IYS becomes more established across Canada,^4^,^9^,^11^,^13^,^17^,^18^ the opportunity to expand and broaden SP in youth wellness services grows.^15^ Local, regional and pan-Canadian IYS networks, such as YouthCan IMPACT,^13^ are implementing models supporting appropriate and timely access to youth well-being services. While substantial evidence supporting the IYS model exists,^9^-^11^ practice-based literature describing model components and service use is needed to facilitate replicability.^11^ Supporting this need, our study adopted a case study methodology to explore how youth wellness hubs engage in social prescribing through service provision. 

***Integrated youth services and social prescribing***

IYS networks in Canada adopt a service hub approach, whereby multiple youth wellness services are most often provided in a single, community space.^9^,^19^,^20^ Hubs commonly address complex social, psychological and physical health needs by providing youth- and family-centred services delivered by integrated care teams using rapid, continuum-of-care approaches in a youth-friendly environment.^2^,^9^ Core to the operation of these hubs are youth and family engagement in service design and delivery, efforts to increase community awareness of services, community network development, measurement-based care and program evaluation activities.^9^ IYS implementation may differ, however, depending on hub context, emphasizing the active involvement of local partners in hub development, including youth, staff, families and external organizations.^9^,^11^,^12^,^21^

SP is a tool that complements mental health and primary care,^22^ and is highly compatible with the IYS model, given that both share the goal of connecting youth to community-based social supports,^18^ which often includes screening for needs and actively supporting access to services.^17^ Moreover, a key pillar of SP in IYS is the notion of person-centredness, in which interventions are designed to empower individuals to improve their own health.^21^ In addition, SP aligns with IYS because both facilitate strong relationships between practitioners by building on pre-existing network strengths, enhancing service tracking and increasing follow-through when working between services.^19^ Evidence supports that challenges with social determinants of health (i.e. access to treatment, food security, employment, education, finances) are common among service-seeking youth,^22^ and models of IYS provide service integrated pathways that address the full range of concerns with which youth are presenting.^9^,^16^

**Reducing health disparities**

Hubs address longstanding issues with system fragmentation by increasing youth access to several different services at once, while providing navigator-supported transitions to higher intensity or other external services if needed.^16^ As multiple service providers become tightly bound within a network, youth benefit from interventions that are more responsive to their current needs.^4^ IYS seeks to remove barriers that commonly prevent youth from accessing timely and appropriate services, such as a lack of trust and awareness.^12^ It accomplishes this by fostering meaningful and ongoing relationships among providers and youth, as well as across the service landscape between organizations and professionals who serve youth.^23^

Research has shown that youth initiatives adopting IYS and SP approaches are successful at reducing health disparities. For example, the Assertive Community Treatment approach integrates rapid and stepped care approaches, including the use of community-based referrals, and has been shown to decrease psychosocial difficulties, as well as depressive and subclinical psychosis symptoms experienced by youth, while improving social interactions and quality of life.^24^ Similarly, an integrated family-based treatment program for adolescents with substance use concerns presenting to community mental health centres included several social prescribing techniques, such as delivering several services at a single localized space and adopting a sequential approach to service provision.^6^ A randomized controlled trial of the program found positive outcomes for youth substance use when compared to treatment as usual.^6^ Finally, integrated behavioural health services using social prescribing techniques have been found to strengthen mental health literacy and commitment to serving youth among practitioners while enhancing practitioner self-efficacy and skill development.^8^

Research and service evaluation in the area of youth-focussed SP is needed to support continued implementation and evidence-based practice. Accordingly, we present a description of SP into a specific IYS model being implemented and evaluated in Ontario, Canada.

**The case study: Youth Wellness Hubs Ontario**

Youth Wellness Hubs Ontario (YWHO) is Ontario’s provincial network of youth-serving hubs that provide integrated services co-designed with youth and families. Currently, there are 22 hub networks with YWHO hubs in 31 geographically diverse communities serving youth aged 12 to 25years. YWHO networks address a continuum of youth needs related to mental health, substance use health, primary care, peer support, navigation, education, employment, housing, wellness activities and other community and social programming.^9^ Available virtually and in person, YWHO hubs are local places where young people have low-barrier, walk-in access to an equity-focussed, high quality, integrated delivery model of support services. Each hub must offer evidence-based or evidence-generating mental health, substance use health, primary care, and social and community supports, though the specific services within each of these domains provided at each hub are determined by local service availability and through consultation and co-development with local youth and community members who form a governance table for the network. 

Also consistent across all hubs is the implementation of youth wellness teams at each location to support enhanced service integration for the clinical service pathway (physical, mental and substance use health services). These teams include mental health and substance use clinicians; medical professionals such as nurse practitioners, primary care providers, and psychiatrists; peer support workers; care navigators; and youth wellness facilitators who support engagement and orientation to measurement-based care. YWHO service pathways comprise a continuum of care for youth, with varying levels of intensity, to facilitate tailoring of services to youth needs, self-reported goals for service, and preferences. 

Reflecting the voices of youth and family members, YWHO services are available to youth without any required referrals, previous assessments or diagnoses. Youth can access services without an appointment (or with an appointment, if preferred), and convenient hours of service (including evenings and weekends) are offered. Youth are also able to move in and out of services with minimal barriers, to reflect their changing needs over the course of development. A full description of the YWHO model, values, and core components has been published elsewhere.^9^

## Methods

***Ethics approval***

This project has undergone ethics review and approval by the Centre for Addictions and Mental Health Quality Projects Ethics Review (#QPER42). 

***Procedure***

This project adopted an embedded case study approach^25^ to profile a novel social prescribing strategy using service data from YWHO hubs. Case studies provide in-depth analyses using a single example of a social phenomenon to highlight more nuanced and novel characteristics,^25^ which are often lost in studies employing multiple case samples. Embedded case studies differ from typical case studies in that researchers are actively engaged in organizational activities and are often a part of the organizational structure (for example, as staff members).^26^ In our study, a descriptive analysis of cross-sectional quantitative service use data was conducted using a large sample of youth accessing YWHO. A comparison of reasons for engaging with YWHO and needs addressed during service provision provide insight into the challenges presented by youth, and how those were addressed in YWHO services. Further, descriptive analyses of services provided illuminate the modalities that were employed with youth. 

***Sample and recruitment***

Participants included youth (n=6361) aged 12 to 25 years receiving services from 14 hubs between April 2020 and March 2023. 

Youth demographic and service data have been collected by YWHO sites since April 2020, and are a routine part of the measurement-based care process between staff and youth that occurs during service visits. At the beginning of a visit, youth are provided with a private space and a tablet programmed to administer measures that include questions regarding needs, goals, symptoms, functioning and demographics. The youth also review and complete a consent form for services that describes the integrated hub services available and the sharing of data among their circle of care. Youth are provided with supports for consent and measure completion as required, but measure completion is not required to access services. Upon completion of the service visit, service providers complete an electronic end-of-visit form, which includes questions about the interventions delivered, needs addressed and next steps in the plan of care. Measures of youths’ experiences and satisfaction with services are also electronically administered. Data are stored in a secure cloud platform, partitioned by site; hub staff can access their own hub’s data. Specific YWHO Provincial Office staff are able to access data across hubs. 

***Measures and analysis***

Data from three variables were analyzed: “reason for visit,” “needs addressed” and “type of service provider” who delivered service. “Reason for visit” is a question completed by youth at the beginning of each visit before services are provided. Youth are asked to indicate the reason they are visiting the hub that day and are provided with several response categories relating to mental, physical, cultural and social needs. “Needs addressed” includes a matching list of needs that is completed by the service provider at the end of the visit to indicate which needs were addressed in session. “Type of service provider” asks service providers to indicate who was involved in service delivery, and is completed at the end of the visit. 

For all variables, multiple responses are allowed. Frequencies for all response categories across each variable were tabulated for service data. Number of selections (n) and percentage of total visits (%) were calculated. Adding further nuance to service data, responses for reason for visit categories were stratified across age and gender demographic variables. This analysis was completed to provide additional information regarding which demographic subgroups were most represented within each service category. Reason for visit was selected because it is the only service variable that is youth-provided, and stratifying additional service variables was beyond the scope of this paper. Analysis was conducted using SPSS Statistics for Windows, version 27.0 (IBM Corp., Armonk, NY, US). 

## Results

***Demographic statistics***

Table 1 provides demographic characteristics of the youth in our sample. The sample is distributed similarly to the whole youth population in Canada.^27^ Response categories for gender, sexual identity, disability status and housing status were collapsed into general categories to increase group size and maintain youth data confidentiality. (Appendix 1 contains a full list of collapsed demographic variables.) Of the sample, 31.8% of youth identified as girl or woman, 25.2% identified as boy or man, and 7.5% identified as trans or gender diverse. For the sexual identity category, 29.7% of youth identified as heterosexual and 19.6% as 2SLGBQI+. For a full list of demographic variables, see Table 1. 

**Table 1:** Demographic characteristics of youth receiving IYS at YWHO hubs, April 2020 to March 2023

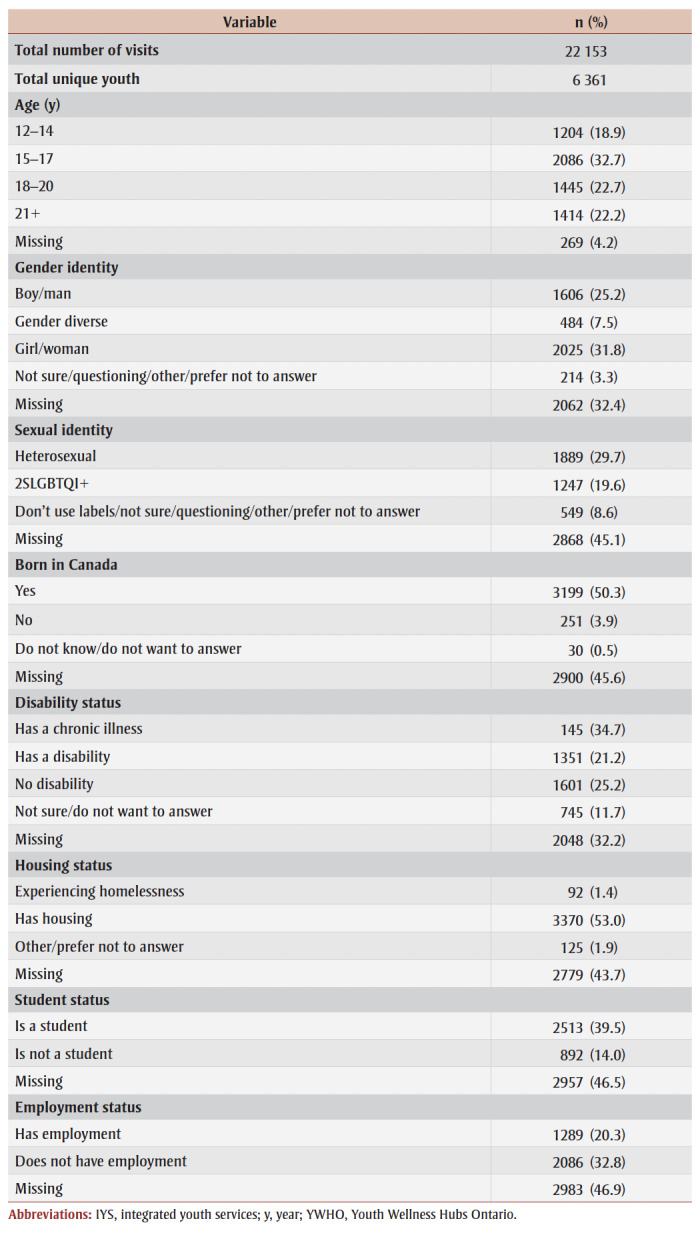

**Appendix 1:** Demographic variables: collapsed categories

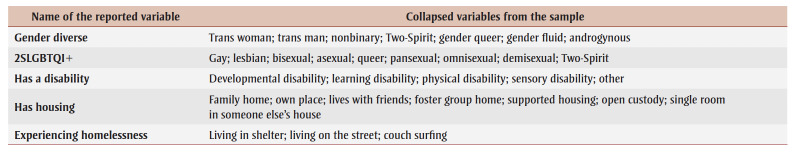

Missing data ranged from 4.2% (age) to 46.9% (employment status). Demographic data is not mandatory for youth to complete (i.e. they may skip providing data for specific demographic questions) but highly encouraged in order that their needs may be best understood. This is an intentional decision, as youth may not feel safe providing identity-based data. Further, response categories are not mutually exclusive, allowing youth to select more than one option per demographic question, in an effort to allow youth to use categories that are most reflective of how they identify. Consequently, counts and percentages for specific demographic and service variables may not be congruous with the sample size. 

***Services requested and provided***

Table 2 summarizes findings from n=6361 youth across 22153 visits (M=3.5 visits/youth) showing youth-reported, pre-service reason for visit and the service provider–reported, post-service needs addressed. Overall, youth were most likely to indicate “mental health” as a reason for visit (47.3%), followed by “school/education” (13.0%), “relationships” (13.0%), “physical health” (6.8%) and “food/nutrition” (5.6%). The remaining categories for reason for visit were less than5.5%. For needs addressed, service providers noted “mental health” was the most common need (72.7%), followed by “school/education” (13.7%), “relationships” (12.1%), “physical health” (8.3%), “substance use” (6.8%) and “employment” (5.7%). All other categories were less than5.5%. Service providers were more likely than youth to select multiple response categories for needs addressed, which had an additional 6942 needs identified (29.5% higher than youth).

**Table 2:** Frequencies of service requests compared to services provided across all visits (n = 22 153) to YWHO hubs from April 2020 to March 2023

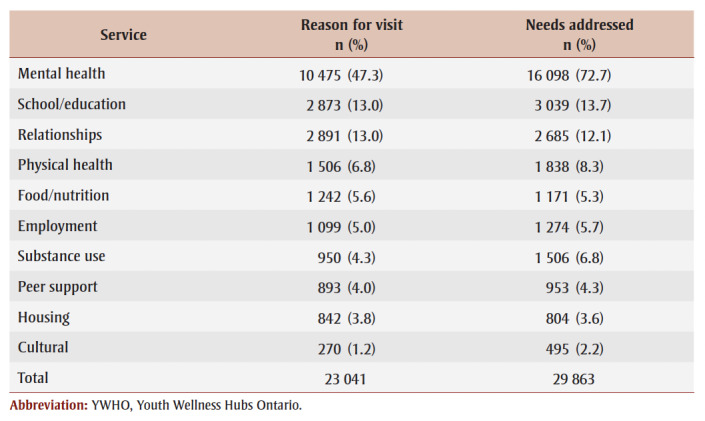

Tables 3 and 4 provide a stratification of reason for visit data across demographic categories for age and gender, respectively. Youth aged 15 to 17 years were the most represented age group in the top three categories, including “mental health,” “school/education” and “relationships.” Frequencies and percentages for other age groups were similar. Youth identifying as “girl/woman” had the highest number of visits for “mental health,” “school/education” and “relationships,” while youth identifying as “boy/man” had more visits for “mental health” than youth identifying as “gender diverse.” Frequencies and percentages for all other categories were similar across gender groups. 

**Table 3:** Reason for visit across age categories of youth visiting YWHO hubs from April 2020 to March 2023

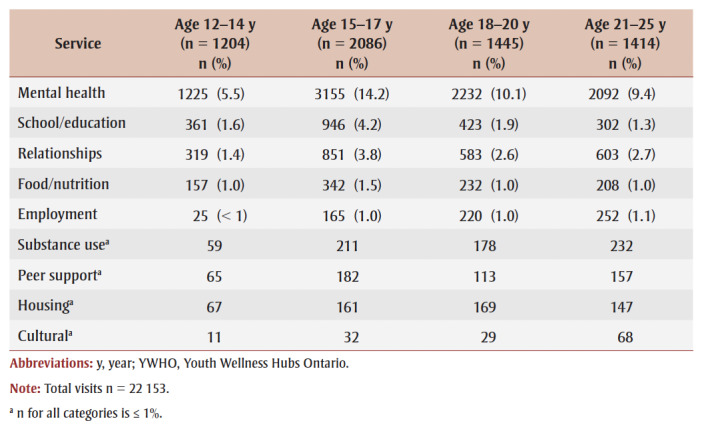

**Table 4:** Reason for visit across gender categories of youth visiting YWHO hubs from April 2020 to March 2023

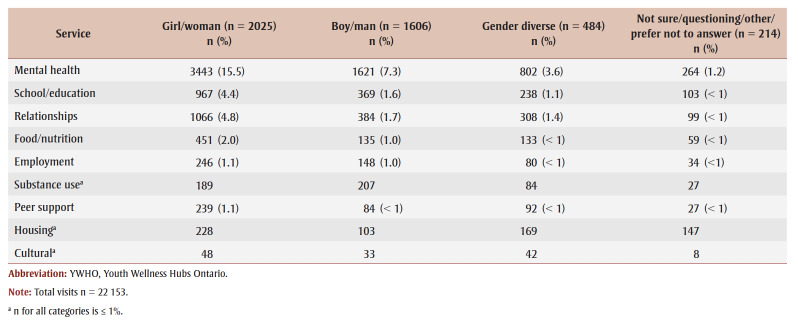

***Type of service provider***

For frequencies of type of service provider (Table 5), the most common service provider was “mental health/substance use clinician/worker” (41.7%), followed by “care navigator/coordinator” (13.4%), “education/training support worker” (6.5%) and “measurement-based care facilitator” (5.7%). All other categories were less than 5%. 

**Table 5:** Frequencies of type of service provider across all visits (n = 22 153) to YWHO hubs from April 2020 to March 2023

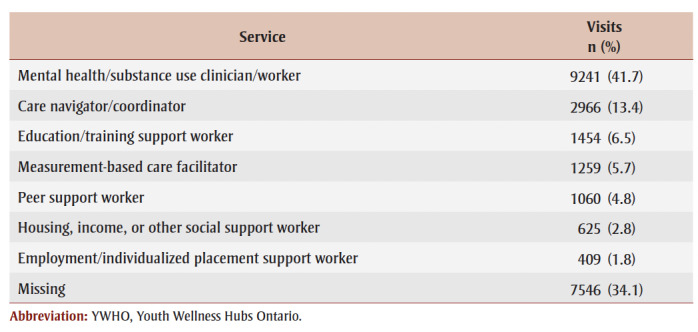

## Discussion

The description of social prescribing within IYS has not been well documented and, in general, the literature lacks in-depth analyses of specific models implementing the SP approach. Using an embedded case study design, we sought to describe an innovative SP model presently being implemented within IYS in Ontario, while analyzing service use data to understand the complexity and intersection of youth needs while engaging in IYS. Our findings provide insight into youth service needs and service delivery while connecting with integrated service hubs. 

With respect to the gender categories, about one-third of youth in the sample identified as girl/woman, reflecting existing research findings that girls and women are more likely to seek support for mental health and other services.^28^,^29^ Transition-aged youth (18–25) represented the largest age group seeking services from YWHO, confirming similar findings across other international models of IYS and further supporting the elimination of typical barriers to help-seeking in this group of young adults.^4^,^22^,^23^

IYS models are designed to address the gaps (e.g. help-seeking barriers, fragmentation, age barriers) that exist in traditional youth mental health and substance use systems, and our data support that young people seeking mental health services will be better served and have improved access where mental health services are offered in integrated settings. 

Youth in this sample selecting mental health as a reason for visit may use this response to indicate the need for several different services within the hub (e.g. group and individual counselling), and the “mental health” reason for visit may also overlap with similar needs, such as peer support, relationships and physical health. Given the association between mental health and the social determinants of health such as finances, employment, food security and housing,^22^,^30^ these data highlight both the need for multidisciplinary professional care in youth services and the ability of YWHO to be responsive to SP needs. These findings also represent further evidence that youth are accessing several different services within hubs that are addressing a wider set of needs, following an SP approach. 

“School/education” and “relationships” were the second most common reason for visit (13%) category. Youth are commonly using YWHO hubs as spaces to complete and access support for schoolwork, highlighting the multifunctionality of hubs and significance to youth as drop-in facilities. The prevalence of “relationships” in service data underscores the importance of interpersonal development for youth, peers and family within mental health services, and how these connections are integral to youth care. Social connection, or lack thereof, is considered a social determinant of health, with documented health consequences, including poor health and socioeconomic status.^31^ SP services have been described as supporting connectedness and, by extension, mental well-being, health behaviours and physical health.^32^,^33^ Integrated services are believed to provide youth with a safe space where various practitioners can address their wholistic needs without youth having to repeat their story multiple times.^34^ In addition, youth can receive services without their peers knowing which services they are accessing.^35^ YWHO has implemented an integrated data platform across its network of service providers that gathers and stores information about the needs, goals and preferences for services of the youth they serve. Service providers who are part of the circle of care for youth have access to their history and can provide personalized measurement-based care. 

Finally, the types of service providers within networks at YWHO hubs highlight the importance of co-location of different service providers who are able to meet the varied needs and goals of youth presenting for service. The fact that care navigator/coordinator was the second most frequently requested service provider (13.4%) highlights the importance of multifunctional care as facilitated by staff who catalyze the SP process in YWHO hubs by navigating health systems, connecting with other providers, completing referrals and ensuring continuity of care. These workers provide youth with valuable supports, ensuring service connectivity and seamless accessibility between services by enhancing communication between providers. Therefore, the role of care navigators and coordinators should be viewed as an essential component of SP and IYS. Similarly, other social support staff are also reflected in the data, and are key for IYS, including education/training, housing, income and employment workers, who together provide over 10% of services. 

***Strengths and limitations***

Several strengths enhance the generalizability of this work. The sample size is considerably large for a study using youth data, as is the number of visits where data were provided. The sample includes representation from several communities, including 2SLGBTQI+ and gender diverse youth. Similarly, data were collected from across the province of Ontario, including large, medium and small population centres. We consider the self-report data to be a strength of this study, as youth voices are often neglected in similar research. This is the first large-scale youth dataset of its kind in Canada. 

However, this study is not without limitations. Data from findings are descriptive, and therefore relationships between variables cannot be ascertained. The absence of inferential analyses prohibits the identification of causal and correlational interactions, and conjecture in the interpretation of findings requires further investigation using multivariate modelling to confirm. The dataset used in this study includes high rates of missing demographic, services provided and service provider data. This was due to the nature of the data collection—most questions provided to youth and staff are not mandatory, and therefore respondents may choose not to provide data for a variety of legitimate reasons (e.g. if a youth is not comfortable providing data, or not in a mental space conducive to providing data on a specific visit). 

Similarly, staff may neglect to complete surveys, either because they have forgotten to complete data entry, or because they have not actually referred a youth to a service. Regardless, missing data may include responses that would alter the nature of findings, although it is not possible to ascertain whether this is the case here. Data validity could have been strengthened in this study by ensuring all variables were responded to by youth. This would ensure continuity across services requested and provided by integrating youth voices throughout. 

## Conclusion

In-depth, descriptive accounts of SP in youth services are largely missing from the knowledge base, but are needed to provide detailed examples of the development, implementation and outcomes associated with related activities. A novel, innovative approach to SP adopted by YWHO embraces IYS as a method for timely and effective referrals across a multidisciplinary set of services addressing youth needs. In our study, a comparative analysis of service data revealed that staff serving youth were more likely to select multiple needs addressed after service delivery, supporting the notion that SP approaches in IYS connect youth to multiple services effectively. A high prevalence of transition-aged youth in the sample lends support to IYS addressing barriers to service access that are common among older youth. Data also show considerable overlap between clinical and nonclinical services, highlighting the need for coordination among multidisciplinary care teams. The diversity of service needs shown in this sample also highlights the importance of effective care navigation. 

Overall, this study frames the case of YWHO as a model for youth SP that may be leveraged to guide other IYS and health service settings. Partners seeking to adopt IYS may consider a similar data collection approach to track service use and identify trends within youth service engagement. Implementation of an IYS system would be enhanced by developing interprofessional care teams to ensure seamless transitions between services addressing the wholistic health, mental health and social support needs of youth. Future research can contribute to the growing body of evidence for IYS by implementing inferential and longitudinal designs that seek to measure change over time across health and mental health outcomes for youth. 

## Acknowledgements

The authors would like to acknowledge the contributions of all members of the Youth Wellness Hubs Ontario Provincial Office past and present, and staff at all YWHO networks. The authors would especially like to acknowledge all of the youth and family members past and present who have contributed to Youth Wellness Hubs Ontario through sharing their enthusiasm, leadership, ideas, time and effort.

## Conflicts of interest

The authors declare there are no conflicts of interest. 

## Authors’ contributions and statement

AT, DC: conceptualization, analysis.

AT, DC: writing—original draft.

MT, JH: writing—review and editing.

The content and views expressed in this article are those of the authors and do not necessarily reflect those of the Government of Canada.
